# A new strategy for the early detection of alzheimer disease stages using multifractal geometry analysis based on K-Nearest Neighbor algorithm

**DOI:** 10.1038/s41598-022-26958-6

**Published:** 2022-12-26

**Authors:** Yasmina M. Elgammal, M. A. Zahran, Mohamed M. Abdelsalam

**Affiliations:** 1 Theoretical Physics Group, Physics Department, Faculty of Science, Mansoura University, Mansoura, Egypt; 2 Computers Engineering and Control Systems Department, Faculty of Engineering, Mansoura University, Mansoura, Egypt

**Keywords:** Biological techniques, Biophysics, Computational biology and bioinformatics, Medical research, Engineering, Physics

## Abstract

Alzheimer's Disease (AD) is considered one of the most diseases that much prevalent among elderly people all over the world. AD is an incurable neurodegenerative disease affecting cognitive functions and were characterized by progressive and collective functions deteriorating. Remarkably, early detection of AD is essential for the development of new and invented treatment strategies. As Dementia causes irreversible damage to the brain neurons and leads to changes in its structure that can be described adequately within the framework of multifractals. Hence, the present work focus on developing a promising and efficient computing technique to pre-process and classify the AD disease especially in the early stages using multifractal geometry to extract the most changeable features due to AD. Then, A machine learning classification algorithm (K-Nearest Neighbor) has been implemented in order to classify and detect the main four early stages of AD. Two datasets have been used to ensure the validation of the proposed methodology. The proposed technique has achieved 99.4% accuracy and 100% sensitivity. The comparative results show that the proposed classification technique outperforms is recent techniques in terms of performance measures.

## Introduction

Alzheimer’s disease (AD) is a widely frequent form of neurodegenerative brain disease^1^. It is responsible for the psychological decline of up to three-quarters of all patients with dementia, which is the major cause of death, leading to a progressive loss of memory and cognitive abilities.

As is well known, the healthy brain has about 100 billion neurons; each neuron has long extensions and branching. These extensions or connections called "synapses" enable neurons to communicate with each other. Through these synapses, signals travel from the presynaptic neuron to the postsynaptic neuron in the form of electrical impulses^2^, causing the release of chemical messages across tiny gaps to the neighboring neurons. Indeed, about 100 trillion synapses are responsible for allowing signals to travel rapidly through the brain’s neuronal circuits, creating the cellular basis of human function, such as memories, sensations, emotions, thoughts, movements, … etc.^3^. Moreover, glial cells play the main role in supporting the function and health of neurons. Microglia, for instance, clear away debris and protects neurons from physical and chemical damage^3^.

**Figure 1:**
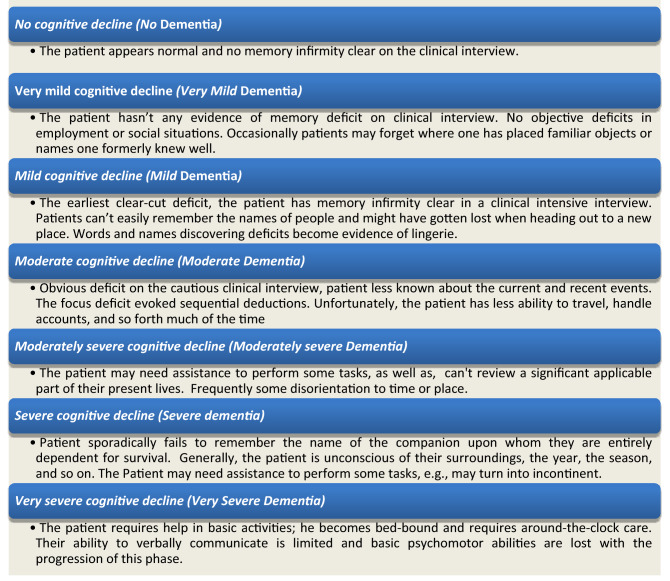
The AD stages according to the global deterioration scale.

However, in a person with AD, changes in the brain are grown due for main two reasons: (1) The protein fragment Beta-amyloid accumulates outside neurons, which clumps into plaques, and (2) protein tau accumulates over time forming tangles inside neurons. The beta-amyloid plaques slowly build up between neurons at synapses, while tau tangles block the transport of nutrients and other essential molecules inside neurons. Eventually, neurons lose their ability to communicate^4^. Moreover, serious irreversible changes occur in the brain, which is believed to set in when the microglia can’t perform their tasks, and atrophy, or shrinking in frontal lobes, temporal-parietal and hippocampus due to cell loss^5^. According to the Global Deterioration Scale (GDS)^6^, the severity of dementia is broken down into seven stages, which predict the primary degenerative of dementia especially AD, and delineation of its stages. These stages can be summarized in Fig. 1:

Alzheimer's disease cannot be diagnosed using a mostly single diagnostic procedure. Physicians employ a number of tools and approaches to aid in the diagnosis, frequently with the help of experts including neuropsychologists, neurologists, geriatricians, and geriatric psychiatrists. To confirm an Alzheimer's diagnosis or rule out other potential causes of symptoms, perform brain scans using magnetic resonance imaging (MRI), computed tomography (CT), or positron emission tomography (PET). Indeed, in some cases especially in the early stages, the assessment might not show Alzheimer's disease, therefore, a doctor might consult to request extra testing. Moreover, the duration between the healthy states to AD spans over many years. At first, the patient suffers from mild cognitive impairment (MCI) and gradually transition to AD. Indeed, not all MCI patients transition to AD^7^. This conversion can be predicted using medical imaging^8^ and other techniques like blood plasma spectroscopy^9^.

The biomarkers for Alzheimer's disease are neurochemical signs that are used to determine whether the illness is present or not. The abnormal deposits of a beta-amyloid (Aβ42), which is considered one of the main causes of the presence of amyloid plaques, the abnormal accumulation of the total tau (*T*-tau) and phosphorylated tau (*P*-tau) in the Cerebrospinal fluid (CSF). The measurement of biomarker levels in the same sample can frequently change dramatically from one institution to another and across various testing platforms, therefore, brain imaging methods are being conducted by several researchers in order to improve diagnosis and progress monitoring.

In this work, we attempt to improve AD classification by combining multifractals features extraction with artificial intelligence, namely the K-Nearest Neighbor algorithm. Therefore, we try to give some remarks and a literature review concerning the above-mentioned terminology.

### Related works

This section has two-fold, i.e., machine learning and fractals analysis. Machine learning^10^ is an application of Artificial Intelligence (AI), where AI techniques seem to be a combination of several research disciplines such as computer science, physiology, philosophy, sociology, and biology. Machine learning aims to extract information from a dataset in order to make a prediction to solve problems related to this data. The application of machine learning techniques gets attention in the classification of AD in several researches in the last decade, see for instance^11–13^.

In recent researches, different machine learning algorithms were applied for developing a predictive model for the classification of AD stages. As in^14^, the authors used recursive feature elimination and applied SVM (support vector machine) to classify several stages as CN versus AD, MCI versus AD, and CN versus MCI, they achieved an accuracy of 100%, 73.68%, and 90% respectively. Also, a developed algorithm called "Support Vector Machine Leave-One-Out Recursive Feature Elimination and Cross-Validation" (SVM-RFE-LOO) for early detection of AD was proposed^15^. Moreover, several researchers also used SVM concerning the detection of AD^16–24^. For instance^25^, used Artificial Neural Network (ANN) with MRI images to perform prediction for the transition from mild cognitive impairment (MCI) to AD with an accuracy of 89.5%. In ^26^, they used the ANN technique to classify AD from cognitively normal (CN) using MRI images with an accuracy of 100%. Another ANN model called the Anatomically Partitioned Artificial Neural Network (APANN) model was used in^27^ in order to predict the clinical score in AD. Chitradevi et al.^28^ used the deep learning technique to classify between CN and AD with an accuracy of 95%.

Deep Neural Network (DNN) learning or Convolutional Neural Network (CNN) was used in several researches. In^29^, a DEMentia NETwork (DEMNET) based on the CNN model was proposed to detect the dementia stages. A modified LeNet model based on DNN was proposed in^30^ using MRI images for AD classification. A volumetric (CNN) model based on MRI images was used in^31^ for multi-classification tasks.

Three classification techniques "Nearest Neighbor, K-Nearest Neighbor, and Weighted K Nearest Neighbor" were used in the detection of AD^32^. The proposed classifiers were used to detect the normal, very mild, mild, and moderate stages with maximum accuracy of 82.67%. A novel feature reduction methodology based on the usage of the KNN classifier was proposed in^33^. The proposed system succeeded in classification into normal, MCI, and AD with an accuracy of 99%.

On the other hand, Euclidean geometry is based on one, two, or three dimensions, which are not realistic in nature. Hence, it is inadequate to approximate the complex and irregular shape of nature within the framework of Euclidean geometry. For instance, the behaviors and structures of the brain system are too complicated to neatly model by traditional Euclidean dimensions. Remarkably, the most vital and significance properties of fractals are self-similar and non-integer dimensions.

Self-similarity falls into three categories: exact self-similarity, at which the fractal is identical at all scales such as the Sierpinski triangle and Koch snowflake^34^. In quasi-self-similarity, the fractal appears approximately identical at different scales. It contains small copies of the entire fractal in distorted forms; for example, the Mandelbrot set's satellites approximate the whole set, but not exact copies. In statistical self-similarity, the structure repeats stochastically as the fractal has numerical or statistical measures, which are preserved across scales, For instance, the brain cells like microglia and astrocytes^34^.

Accordingly, fractals fall into two categories: mono-fractal and multifractal concerning non-integer dimensions. This fractal dimension plays an important role to quantify how the fractal structure fills the space under consideration. In other words, fractal dimension (FD) is an index that describes the fractal properties e.g. this index measures scale-invariant details. In nature, one exponent (FD) is not sufficient to describe the complexity of different patterns, such as human physiology. Comparatively, multifractal geometry offers a spectrum of FDs, which can be deemed as a superposition of homogeneous exact self-similar fractals and is more appropriate for analyzing such complexity.

Therefore, the utilization of fractal geometry in neurosciences has been the outcome of a new trend of research focused on the analysis of the complexity of biological systems^35–41^. The application of the fractal dimension (FD) in investigating the clinical-pathological spectrum of neurodegenerative diseases including AD^42^. Smits et al.^43^ extracted Higuchi’s fractal dimension (HFD) from resting-state eyes-closed electroencephalography (EEG) to show the sensitivity of HFD to brain activity changes in CN and AD. The FD changes in a cross-sectional cohort of patients with AD and front temporal dementia (FTD) were estimated, giving distinct that the cortical complexity relates to cognitive domains impairment^44^. In^45^, the authors used fractal analysis in MRI images to study the changes in the brain due to AD. Both FD and lacunarity were measured for the detection and diagnosis of neurodegenerative diseases, including AD. In^46^, they investigated the temporal-scale-specific fractal properties, and then compared the values of the temporal-scale-specific fractal dimension between CN and AD patient. Peng Li et al.^47^ showed that fractal regulation (FR) could predict AD as they assessed FR in motor activity, which was degraded in dementia.

Multifractal dimensions were used for detecting AD in the mild stage based on SVM for individual and multiple kernel learning (MKL) for combined features^48^. The maximum classification accuracy reached 76%. Another research^49^ used multifractal features for differentiating the Early Mild Cognitive Impairment (EMCI) from other Alzheimer’s disease stages. The classification is based on demonstrating the variation of singularity spectrum function *f* (α). The classification accuracy reached 97% using the SVM classifier. Several researchers used multifractal in medical image analysis^50–52^.

Therefore, the main motive of this research is to propose a novel computational method to automatically classify various stages of Alzheimer's Disease based on the utilization of multifractal geometry analysis. The methodology is based on extracting the multifractal features that are related to changes in the brain structure due to atrophy. The classification system uses a simple K-Nearest Neighbors technique (KNN) for detecting the early four stages of AD (no cognitive decline, very mild cognitive decline, mild cognitive decline, or moderate cognitive decline). To verify the effectiveness of the proposed technique, two different datasets have been used, as well as, a comparative study with the recent techniques has been included. The results show that the proposed methodology has improved the performance measures.

### The research contributions

The summary of the previous discussion can be written in the following points:

1. The classification techniques such as CNN, DNN, and ANN require a large number of images as training, validation, and testing datasets. As well as, large time-consuming for training and testing.
2. The traditional or modified classifiers as SVM, KNN, weighted KNN, … etc. Some of these methodologies achieved moderate performance measures, others were based on the features that were already extracted in a parameter-data file by the owner of the dataset, and some detect one to three stages of AD.
3. The multifractal geometry can be used in describing the morphological changes in the brain image according to the selected parameters, which is the state of the art of the researchers' methodologies. Unfortunately, the multifractal analysis can't be used alone as a discriminant tool because it is a describing or analysis tool. Therefore, some methodologies in the detecting of some diseases are based on comparing the different stages of the disease together for the ease of demonstrating them, rather than determining the identity of the stage directly without resorting to comparisons with other stages.
4. Choosing the appropriate multifractal parameters will remain the state-of-the-art methodology, which may affect the system's accuracy.
5. To our knowledge, there is no sufficient research on analyzing medical images using multifractal geometry integrated with machine learning techniques.

Therefore, the contribution of this research can be summarized as:

1. Use multifractal geometry as an analysis tool to extract the most features related to the changes in the brain structure for the AD classification into the early four stages. As Multifractals enable feature reduction compared with alternate extracting features algorithms.
2. The methodology can discriminant the raw image into the specific stage directly without comparing it with other stages.
3. Two different datasets have been used in order to ensure the effectiveness of the proposed methodology.
4. The proposed methodology has achieved 99.4% accuracy and 100% sensitivity.

## Materials

The working Alzheimer's dataset images were collected from two different sources. The first source is Kaggle international data science community^53^. The total working dataset images were 560 MRI images, 460 subject images for constructing the classification technique, and 100 images for testing. The 460 subject images were classified into 140 subject images for each no cognitive decline, very mild cognitive decline, mild cognitive decline, and moderate cognitive decline. The second source is the ADNI database^54^. The ADNI database contains a T1 weighted MRI image with 1.5 T. The total used images were 750 MRI images comprising 200 CN, 200 MCI, 200 AD, and 150 for testing.

**Figure 2:**
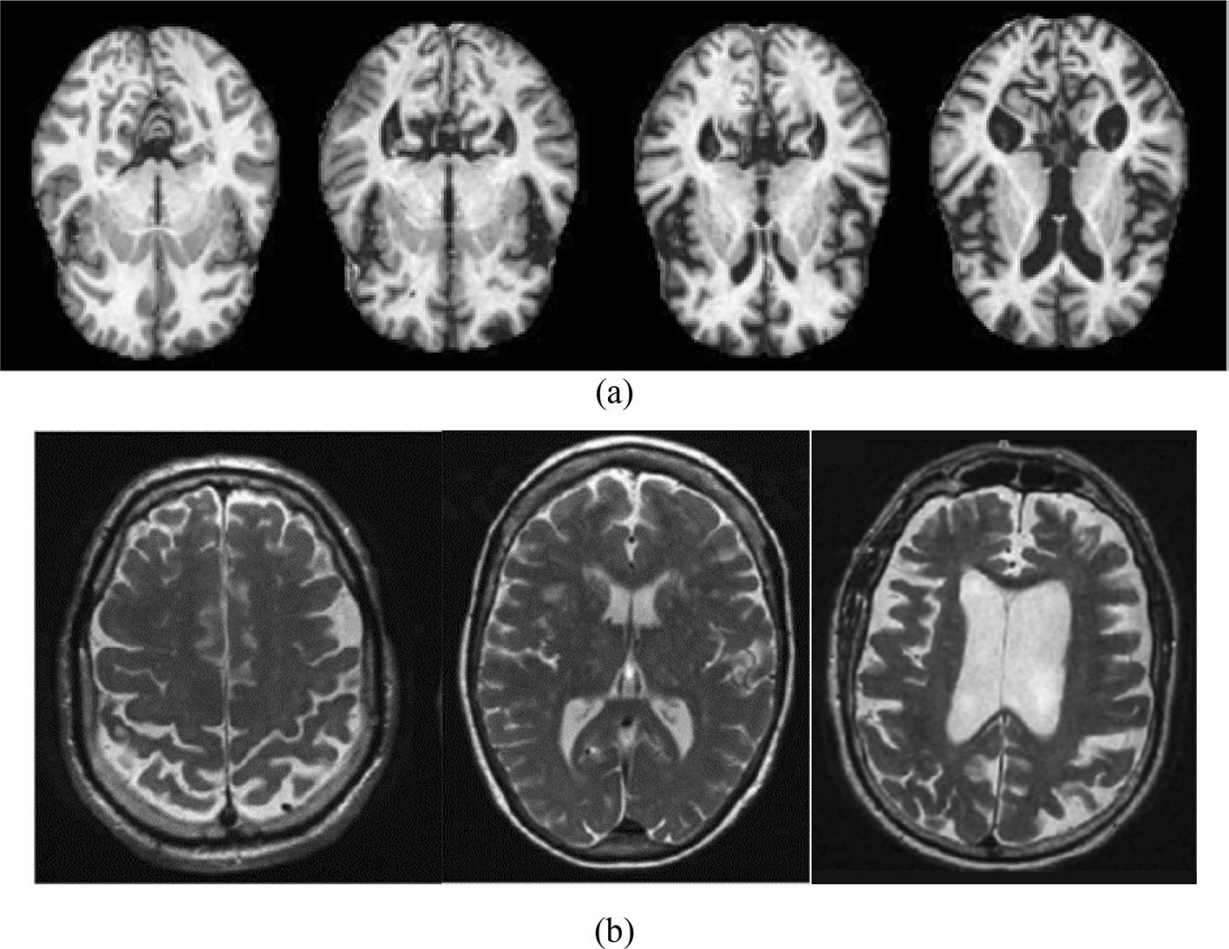
The differences in the area of CSF in (**a**) the kaggle dataset the images from left to write as no dementia, very mild dementia, mild dementia and moderate dementia (**b**) the ADNI datasets the images from left to right as CN, MCI and AD.

In this research, image preprocessing is the first stop of the classification process. The images have been processed for resolution and contrast enhancement, which enables the detection of the changes in the area of cerebrospinal fluid (CFS) in the brain as shown in Fig. 2. Due to the atrophy occurring in the patient brain, the CFS area increases with the progress of the disease. Figure 2 shows different images according to the used dataset. All images, despite the difference in the dataset, have a general feature, which is a shrinkage of the brain with an increase in the CFS area according to the disease stage.

## Methods

### Multifractal analysis

**Figure 3:**
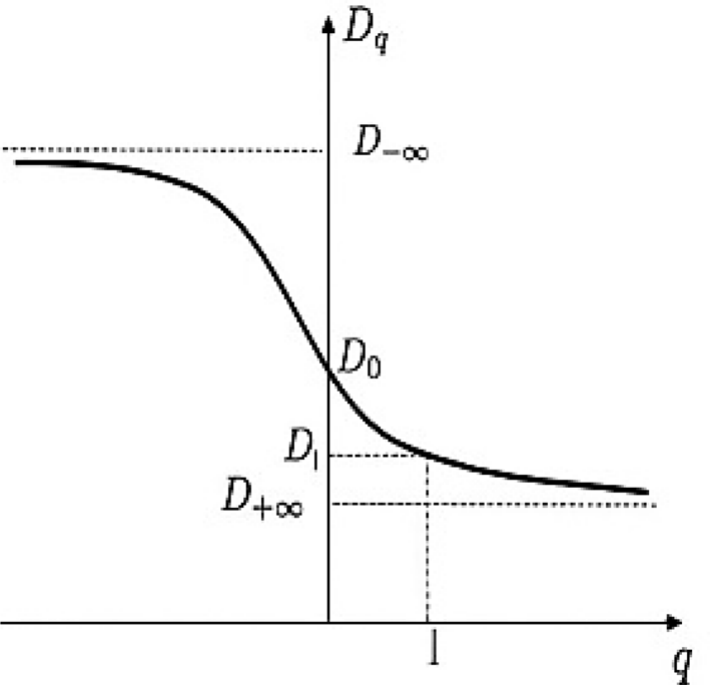
The multifractal generalized dimension.

In fractal geometry, the occupation of the area is very wide structures in a small volume, as the brain geometry, provides a high degree of interconnectivity in a very small volume. The multifractal analysis identifies patterns characterized better by a spectrum of FDs than a single FD. In this case, researchers applying warping filters to the image are used to illustrate features that are unnoticeable. These warp filters are a set of exponents denoted by the symbol (*q*). For each *q*, one can determine the generalized dimension (Dq), as in Fig. 3. Also, it can easily see that the curve becomes generally steeper slopes around *q* = 0 for multifractal structures.

#### Generalized dimension

The generalized dimension D_q_ can be defined as:

1 $$ D_{q} = \frac{1}{1 - q}\mathop {\lim }\limits_{r \to 0} \frac{{\ln I\left( {q,r} \right)}}{{{\text{ln}}\left( {1/r} \right)}} $$

with I(q,r) is the partition function given by:

2 $$ I\left( {q,\;r} \right) = \ln \sum\nolimits_{i = 1}^{N\left( r \right)} {P_{i} \left( r \right)^{q} } $$

Insert (2) into (1), yields:

3 $$ D_{q} = \frac{1}{1 - q}\mathop {\lim }\limits_{r \to 0} \frac{{\ln \mathop \sum \nolimits_{i = 1}^{N\left( r \right)} P_{i} \left( r \right)^{q} }}{{{\text{ln}}\left( {1/r} \right)}} $$

where, r denotes the scale of measurement, q is the order of moment, *N*(r) the number of fractal copies based on the scale *r*, and *P*_i_(r) is the growth probability function of the *i*^th^ fractal unit. From the general dimension definition, one can derive three fractal dimension concepts:

- Box counting dimension (D_B_)
- Information dimension (D_I_)
- Correlation dimension (D_C_)

These dimensions represent three basic parameters of fractal spectrums. Let us start with:

1. Box-counting dimension:

The box-counting method is based on covering the object with a small cell of definite size. At *q* = 0, D_o_ describe the box-counting dimension D_B_, which is known also as the capacity dimension. In Eq. (3), when we use a grid of boxes to cover a given space, the box-counting dimension D_0_ can be written in the following formula:

4 $$ D_{0} = \mathop {\lim }\limits_{r \to 0} \frac{\ln N\left( r \right)}{{\ln \left( {1/r} \right)}} $$

when, N(r) is the number of nonempty boxes with length r that cover the space and include at least some part of the attractor (not necessarily the total number of points).

2) Information dimension:

At *q* = 1, D_1_ is known as the information dimension that characterizes the rate of information loss by the time or the rate of information gain by sequential measurements. *D*_1_ in essential to a quantity known as the Shannon entropy. Shannon entropy is the measure of the average information when the value of the random variable is unknown. It is defined as:

5 $$ H\left( r \right) = - \sum\nolimits_{i = 1}^{N\left( r \right)} {P_{i } \left( {\text{r}} \right){\text{ ln}}P_{i} \left( r \right)} $$

then, apply the Taylor expansion to Eq. (2), one finds:

6 $$ \ln I\left( {q,\;r} \right) = \left( {q - 1} \right)ln\sum\nolimits_{i = 1}^{N\left( r \right)} {P_{i} \left( r \right)\ln P_{i} \left( r \right)} $$

So, Eq. (3) becomes:

7 $$ D_{1} = \mathop {\lim }\limits_{r \to 0} \frac{{\ln \mathop \sum \nolimits_{i = 1}^{N\left( r \right)} P_{i} \left( r \right)\ln P_{i} \left( r \right)}}{{{\text{ln}}\left( {1/r} \right)}} $$

3) Correlation dimension:

At *q* = 2, *D*_2_ is the correlation dimension, which characterizes the correlation between pairs of points on a reconstructed attractor. From Eq. (3), the correlation dimension *D*_2_ can be described as:

8 $$ D_{2} = \mathop {\lim }\limits_{r \to 0} \frac{{\ln \mathop \sum \nolimits_{i = 1}^{N\left( r \right)} P_{i} \left( r \right)^{2} }}{lnr} $$

It worth mentioning that, If *D*_0_ = *D*_1_ = *D*_2_, the structure is termed as mono-fractal or fractal. However, in the case of *D*_o_ > *D*_1_ > *D*_2_, the structure then is termed as multifractals.

#### Singularity spectrum

**Figure 4:**
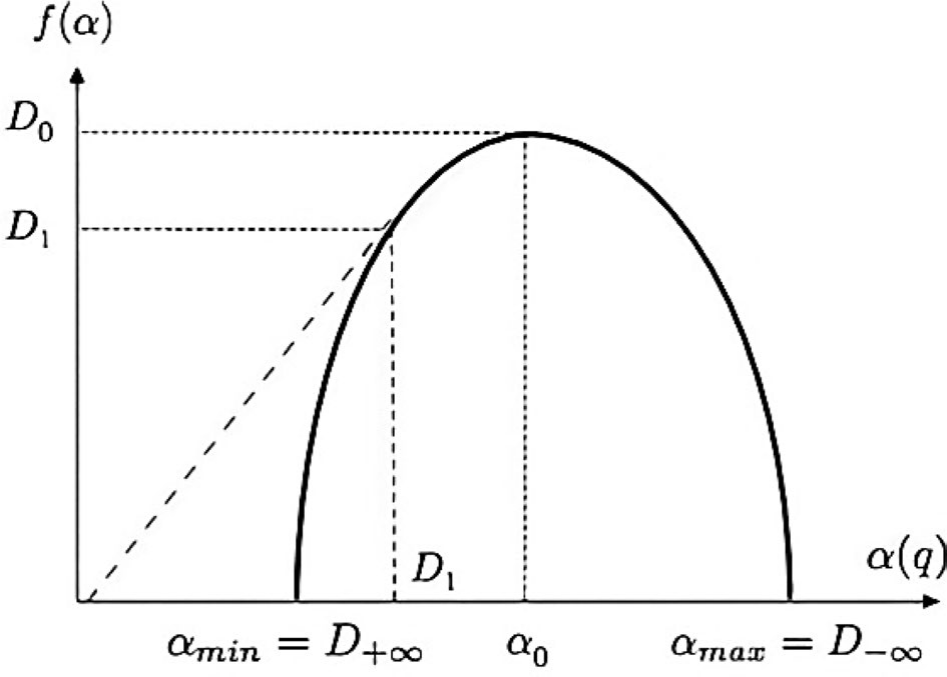
The *f* (*α*) spectrum curve.

Singularity spectrum *f (α)* is another description of the multifractal spectrum, which involves analyzing fractal measures into combination sets, each of which is characterized by its singularity exponent *α* and its fractal spectrum *f (α)*. Indeed, singularity spectrum *f (α)* relates to the generalized dimensions *D*_q_, which can be written as:

9 $$ \tau \left( q \right) = \left( {1 - q} \right)D_{q} $$

where, *τ (q)* denotes the mass exponent of multifractal structure. By employing Legendre transformation, *τ (q)* and *D*_q_ can be converted into a pair of local parameters of multifractals:

10 $$ \alpha \left( q \right) = \frac{d\tau \left( q \right)}{{dq}} = D_{q} + \left( {q - 1} \right)\frac{{dD_{q} }}{dq} $$

11 $$ f\left( \alpha \right) = q\alpha \left( q \right) - \tau \left( q \right) = q\alpha \left( q \right) - \left( {q - 1} \right)D_{q} $$

with *f* (α) denotes the fractal dimension of the fractal units of certain sizes, and *α* (*q*) is assumed as the corresponding singularity exponent. The spectrum curve can be shown in Fig. 4. Concerning any particular spectrum curve, the right of its maximum corresponds to *q* < 0 and the left to *q* > 0. Remarkably, compared this curve to mono and non-fractals, multifractal are characterized by broader *f* (α) curve.

### The K-Nearest Neighbor algorithm

**Figure 5:**
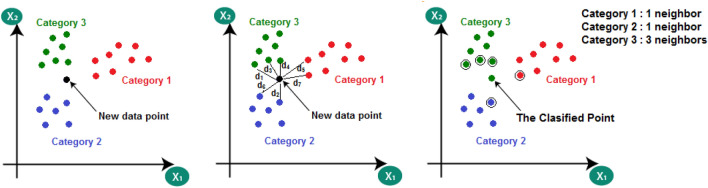
The KNN algorithm.

The KNN algorithm is one of the widely used machine learning based on the supervised learning technique. It can be used to solve both classification and regression problems^55–57^. The most usage is in the classification technique. It can classify the input datasets into multiple categories. The main idea is based on storing the available datasets, then classifying the new data according to the nearest or similar stored dataset this can be summarized in Fig. 5.

In the first step, the datasets were located in the plane according to the number of features as shown in Fig. the data has two features × 1 and × 2. Then according to the new data point, all distances (d_1_, d_2_, d_3_, …, d_n_ where n is the number of the raining data samples) were calculated from the data point to all training data points. Finally, for an effective value "K", select the minimum k data point distances and the classification was done according to the nearest neighbor as shown in Fig. 5.

Therefore, the KNN algorithm has the advantages of simple implementation, robustness especially in noisy training data, no need for training procedure, and effectiveness in large training data sets. Although, the determination of the selective K value sometimes is complex. As well as, the high computation cost due to the distance calculation between the data points and all other training data samples.

In this study, Alzheimer's disease stages can be classified using the KNN algorithm. The objective of the proposed system is to detect and classify the AD stages into four stages non- dementia or normal, very mild-dementia, mild-dementia, and moderate-dementia in case of using the Kaggle dataset or three stages CN, MCI, and AD in case of using ADNI dataset. The classification technique is based on extracting the most ten changeable features of the brain geometry using multifractal analysis. These features can be listed as:

1. *D*_1_ is the information dimension.
2. *D*_2_ is the correlation dimension.
3. The local dimension at the maximum singularity spectrum curve (*α*_0_).
4. The minimum local dimension (*α*_min_).
5. The start value of the singularity spectrum (*f(α*_min_))
6. The maximum local dimension (*α*_max_).
7. The end value of the singularity spectrum (*f(α*_max_))
8. The width of the singularity spectrum (*W*)
9. The symmetrical shift of the singularity spectrum curve.
10. The apparent area of the brain section in image (A).

**Figure 6:**
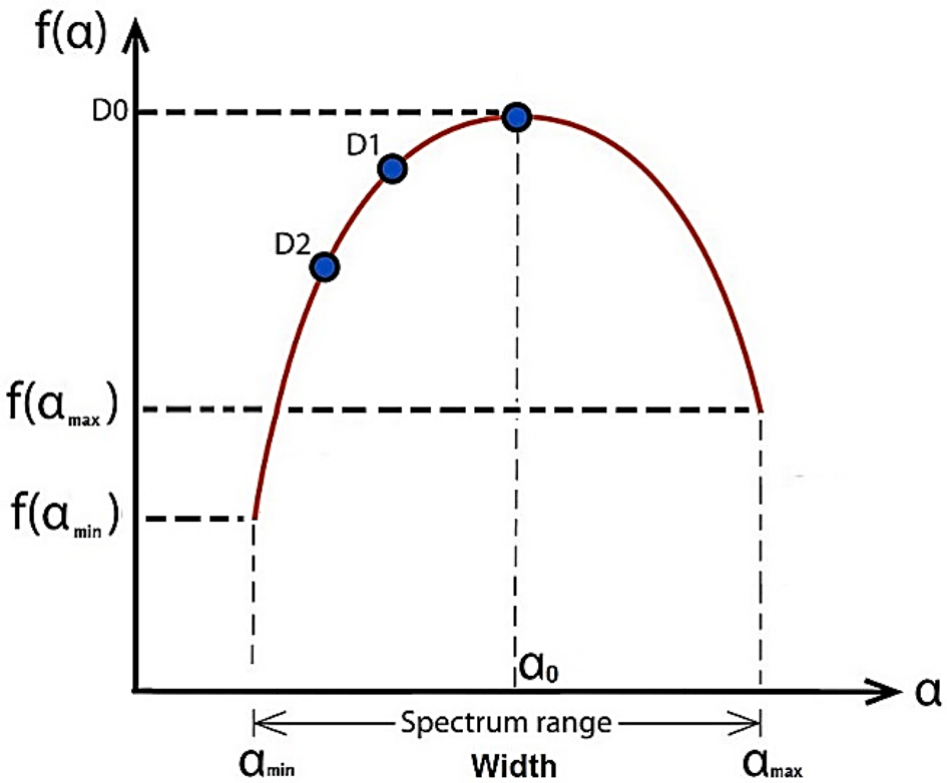
The extracted features.

These features are shown in Fig. 6

### Case study

From the previous discussion, Alzheimer’s disease causes many cellular and molecular changes in the brain. These changes can be summarized as: (1) disturbance of the cell functions due to the abnormal levels of the beta-amyloid protein that clumps to form plaques that collect between neurons. (2) The abnormal accumulations of tau protein that collect inside neurons cause neurofibrillary tangles that block the neuron’s transport system. (3) Chronic inflammation caused by microglia that fail to clear away beta-amyloid plaques, waste, and debris in the brain. (4) Vascular problems due to the deposition of beta-amyloid in brain arteries. (5) Losing the neural connections.

All these changes lead to: (1) a change in the brain structure, (2) loss of neurons, and (3) the texture information, volume, and shape of the white matter, gray matter, and hippocampus. Therefore, one obtained a new brain structure that can be described using multifractal geometry. Hence, multifractal geometry can describe gaps and their distribution, the brain volume or area, matter distribution, texture information, and the brain structure's heterogeneity.

**Figure 7:**
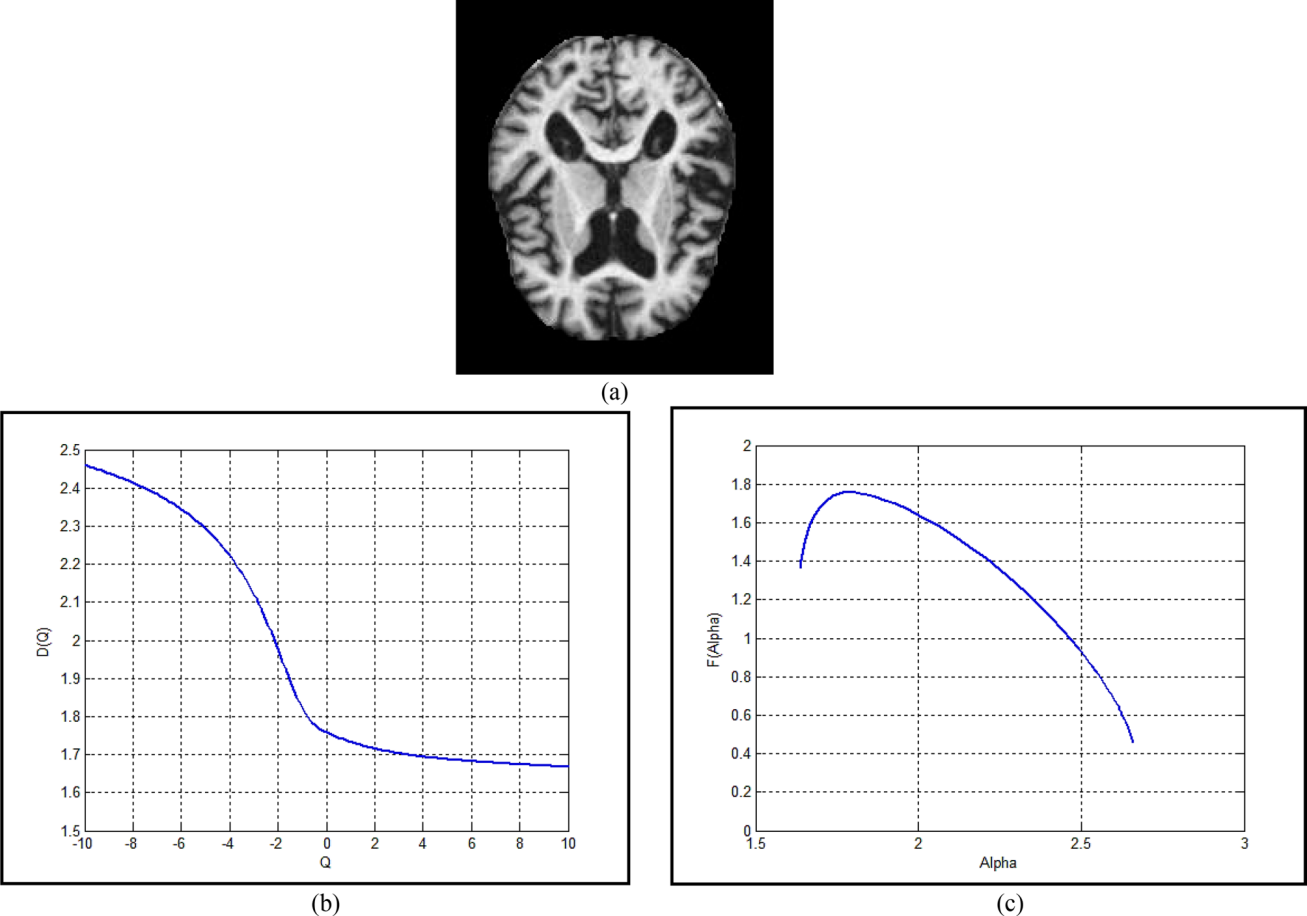
The brain sample image (**a**) the source image (**b**) the generalized dimension curve (**c**) the singularity spectrum.

Let us illustrate the previous concepts with a brain image example. Figure 7(a) shows a sample image of Alzheimer's disease at a moderate dementia stage. The source image may have a moderate or little resolution according to the imaging process or the available data, therefore, the first step is to enhance the resolution and the contrast of the brain image this can be achieved by a custom-written MATLAB program. The multifractal analysis results can be obtained as shown in Fig. 7b,c. Figure 7(b) shows the generalized dimension of the brain sample image. The information dimension *D*_1_ = 1.73 means the brain image has some morphological changes due to more deposits of a beta-amyloid causing amyloid plaques, as well as the (*T*-tau) accumulation that lead to the brain shrinking. The correlation dimension iD_2_ reflects the correlation between pair of pixels in the scanning box, at *D*_2_ = 1.715 for the given sample image, which means the pixels are not contiguous, more gaps appeared in the image due to cell loss and more shrinking in frontal lobes, temporal-parietal, and hippocampus. The singularity spectrum at Fig. 7c has the following features: (1) Broader spectrum, Fig. 7c has wide range of variability starting from *α*_min_ = 1.673 to *α*_max_ = 2.66 with a width = 0.987. That confirms the presence of many gaps and atrophy of multiple dimensions and sizes distributed on the lobes of the brain. (2) Asymmetric curve, as the singular spectrum is characterized by an asymmetric curve, hence the center should be at α_0_ (in this case study *α*_0_ = 1.78) which is close to αmin making a shift by 0.386 to the symmetrical axe. That related to the heterogeneity zones that appeared in the brain structure. (3) High variability between starting value of the singularity spectrum *f*(*α*_min_) = 1.36 and the ending point *f*(*α*_max_) = 0.452, which means the brain structure image has heterogeneous between its lobes.

**Figure 8:**
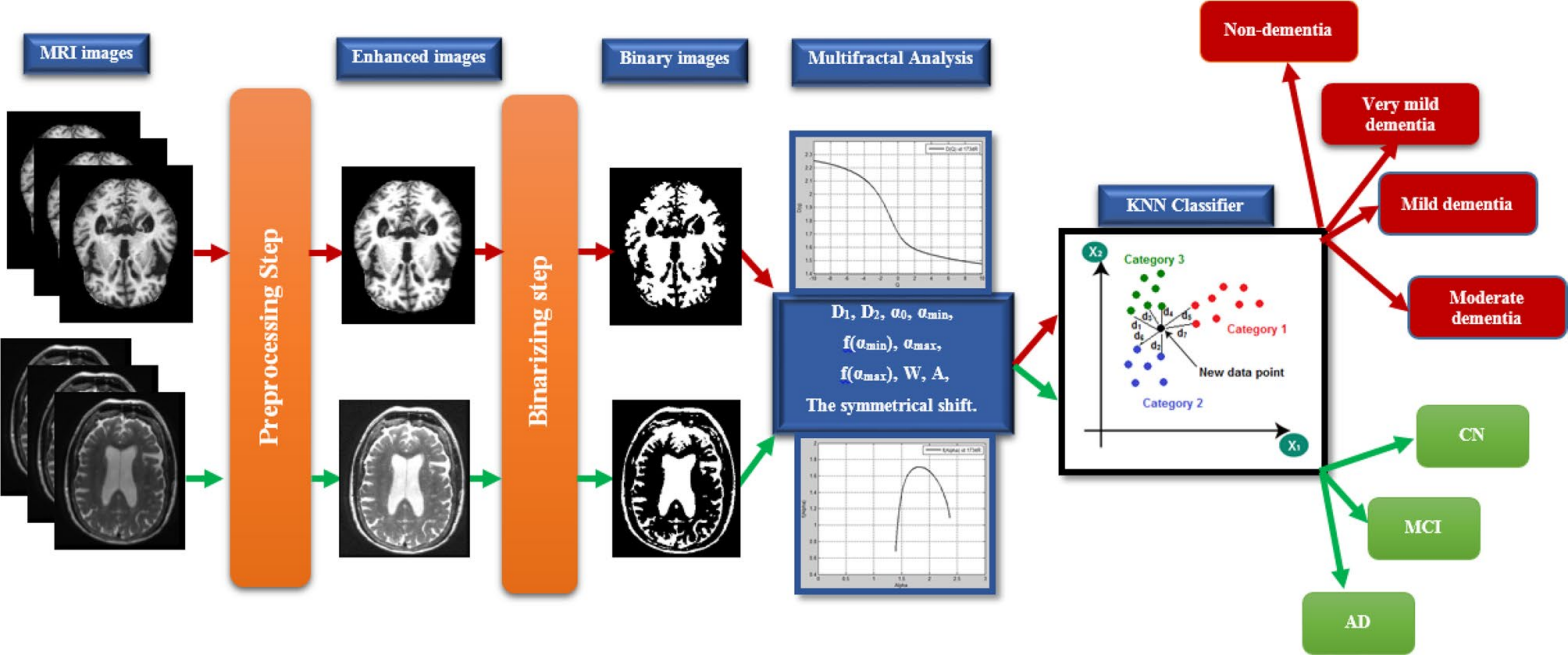
The workflow of the proposed methodology.

According to the previous discussion, the flow chart of the proposed methodology is illustrated in Fig. 8. The methodology can be summarized as.

1. The raw images are used from two image sources as Kaggle dataset and the ADNI dataset. A custom-written program using MATLAB software for preprocessing the raw images as enhancing the contrast and resolution of the input images.
2. Binarizing the resulting images means converting the images after the preprocessing step into black and white images according to an empirically pre-defined threshold.
3. Using multifractal analysis to extract the ten changeable features related to the brain structure changes.
4. Apply the KNN to the resulting features to classify the raw image into non-dementia or normal, very mild-dementia, mild-dementia, and moderate-dementia in the case of the Kaggle dataset, or CN, MCI, and AD in the case of ADNI dataset

## Results and discussion

### The demographic characteristics

In this research, 400 MRI brain images have been analyzed. The images are categorized as 100 images for normal, 100 images for very mild, 100 images for mild, and 100 images for moderate patients as obtained from the available online Kaggle challenge. As well as, 150 images for CN, 150 for MCI and 150 for AD from the ADNI dataset. All subjects have aged over 65 years. The demographic characteristics are shown in Table 1.

**Table 1:** The demographic characteristics for Alzheimer's disease subjects.

Item	Kaggle dataset				ADNI dataset		
	No dementia	Very mild dementia	Mild dementia	Moderate dementia	CN (Cognitively normal)	MCI (Mild cognitive impairment)	AD
Training data	100	100	100	100	200	200	200
Testing data	40	40	40	40	50	50	50
Total	140	140	140	140	250	250	250
Female/Male	65/75	69/71	68/72	66/74	125/125	125/125	125/125
*p* value	0.1751				1.000		
	560 MRI images				750 MRI images		

Statistics are used to analyze data from Table 1 to determine the significance of the demographic characteristics. The data are considered significant for this study if the corresponding *P* value is less than 0.05 (*P* < 0.05). The female/male subjects are 268/292 with *P* = 0.1751 (*P* > 0.05). Therefore, the demographic characteristics are statistically non-significant.

### Image analysis using multifractal geometry

**Figure 9:**
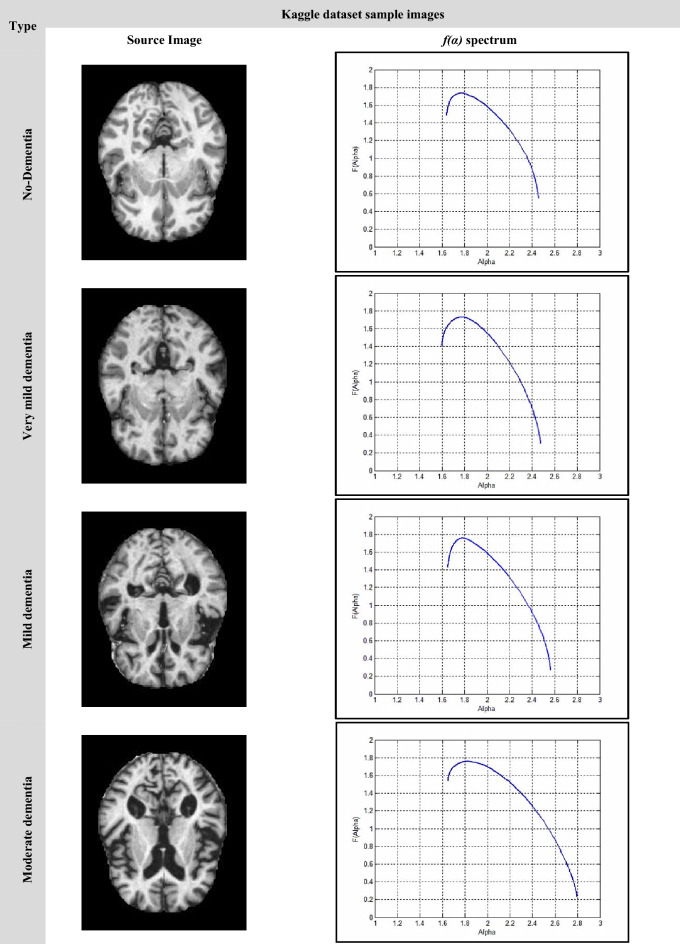
The Kaggle dataset sample images.

**Figure 10:**
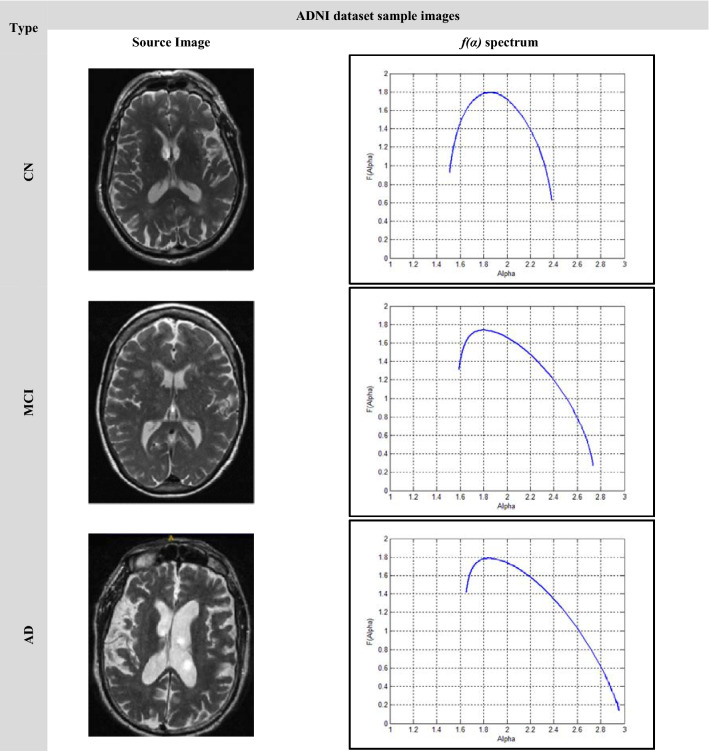
ADNI dataset sample images.

Image samples of a single brain slice for different stages of the used datasets are shown in Fig. 9 and Fig. 10. Figure 9 illustrates the *f(α)* spectrum for the different stages of AD. According to the progression of the disease with more deposits of beta-amyloid and tau proteins, more amyloid plaques were found causing brain trophy. The more changes in the structure of the brain and its shrinkage, the more the multifractal parameters change, and this is shown by shifting the spectrum to the right and increasing its variability and width. Increasing the difference between the starting and ending values of the singularity spectrum *f*(*α*_min_) and *f*(*α*_max_) respectively. Similar behavior can be shown in Fig. 10 with the ADNI dataset. In the CN stage, the singular spectrum tends to be a symmetric and narrow curve for no abnormalities found in the brain structure. While in MCI and AD stages, the spectra lose their symmetric shapes, as well as they, shift to the right with increasing change in the multifractal parameters.

**Figure 11:**
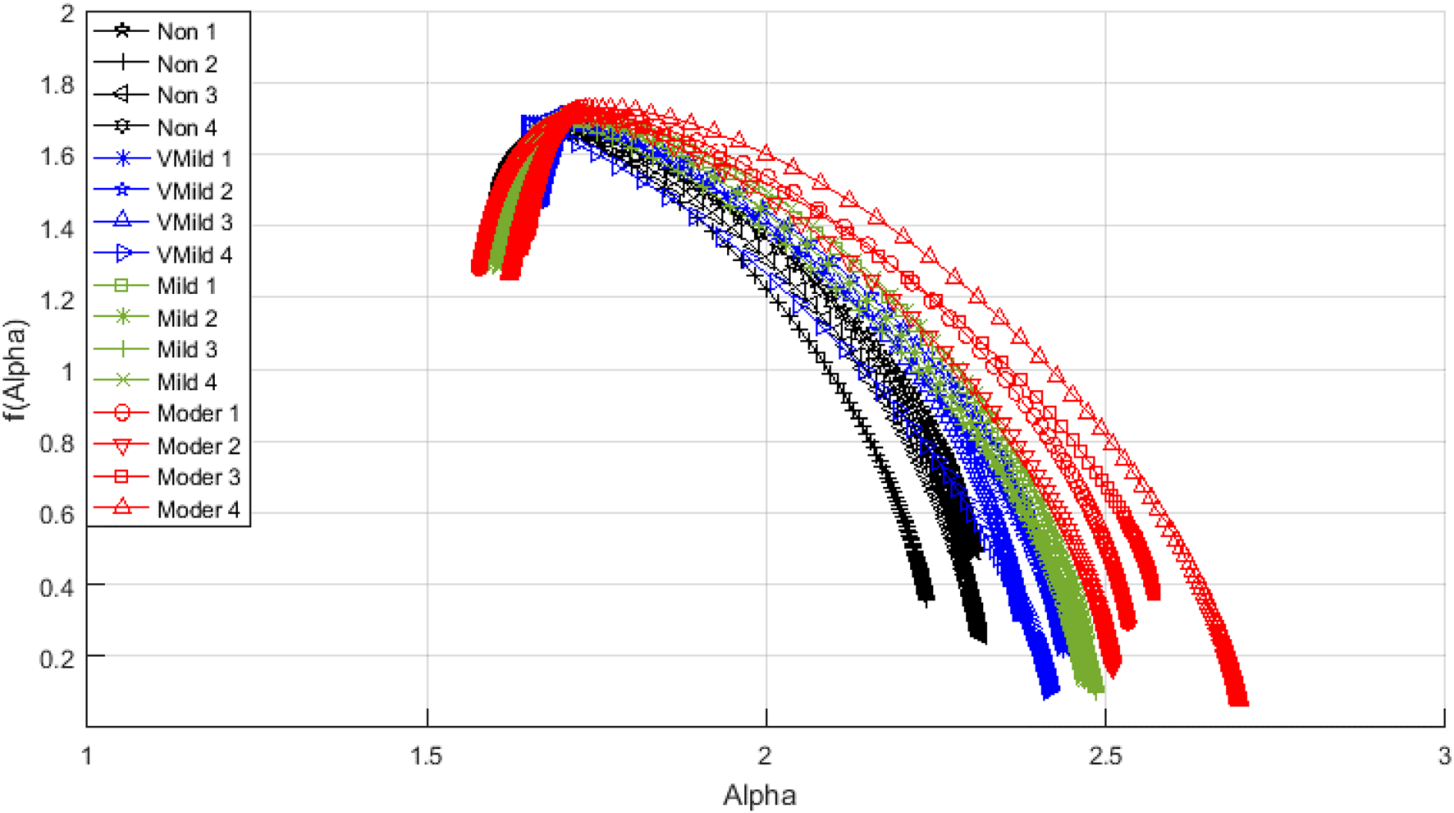
The singularity spectra for the AD stages (16 sample images-Kaggle).

**Figure 12:**
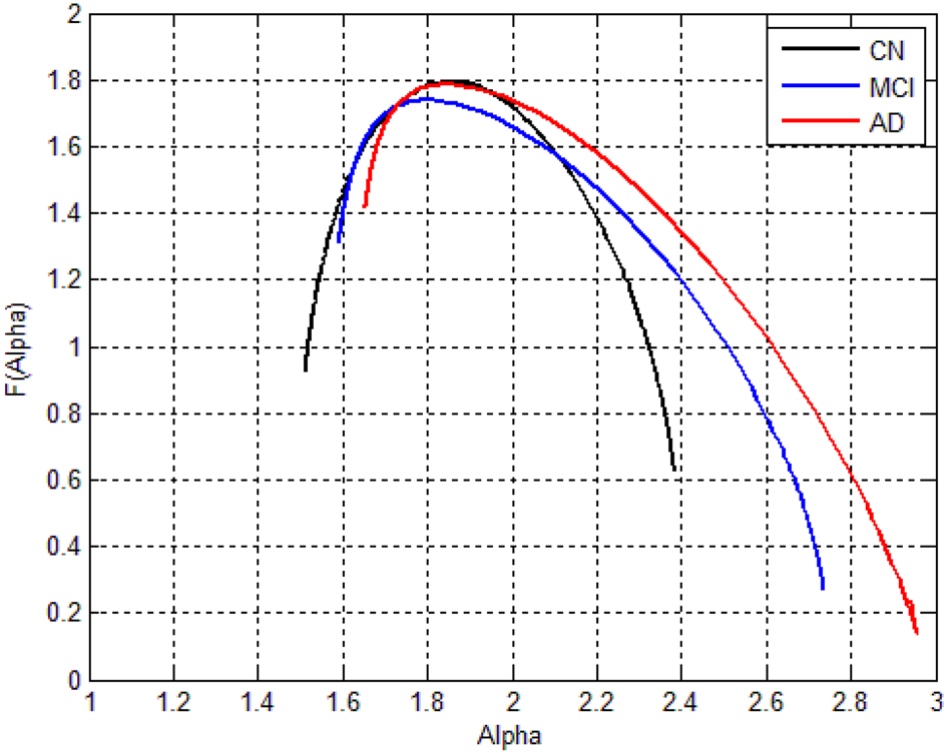
The singularity spectra for the AD stages (3 sample images-ADNI).

In order to ensure the ability of the multifractal geometry in describing the complex structures for example the changes in the brain structure, a set of comparative spectra representing the different AD stages can be shown in Figs. 11 and 12. As shown in Fig. 11, the singularity spectrum has changed according to the AD stages. The maximum local dimension (αmax) has reached its minimum value in the normal cases with 2.25, while the maximum value has been achieved in moderate cases with 2.8. As the AD disease progresses, the spectrum is broader and shifts to right.

In the case of the ADNI dataset as shown in Fig. 12, there is a clear contrast between the different stages of Alzheimer's disease, which confirms that, the significance of the multifractal parameters that were chosen to describe the spectrum or in other words the brain structure changes.

**Figure 13:**
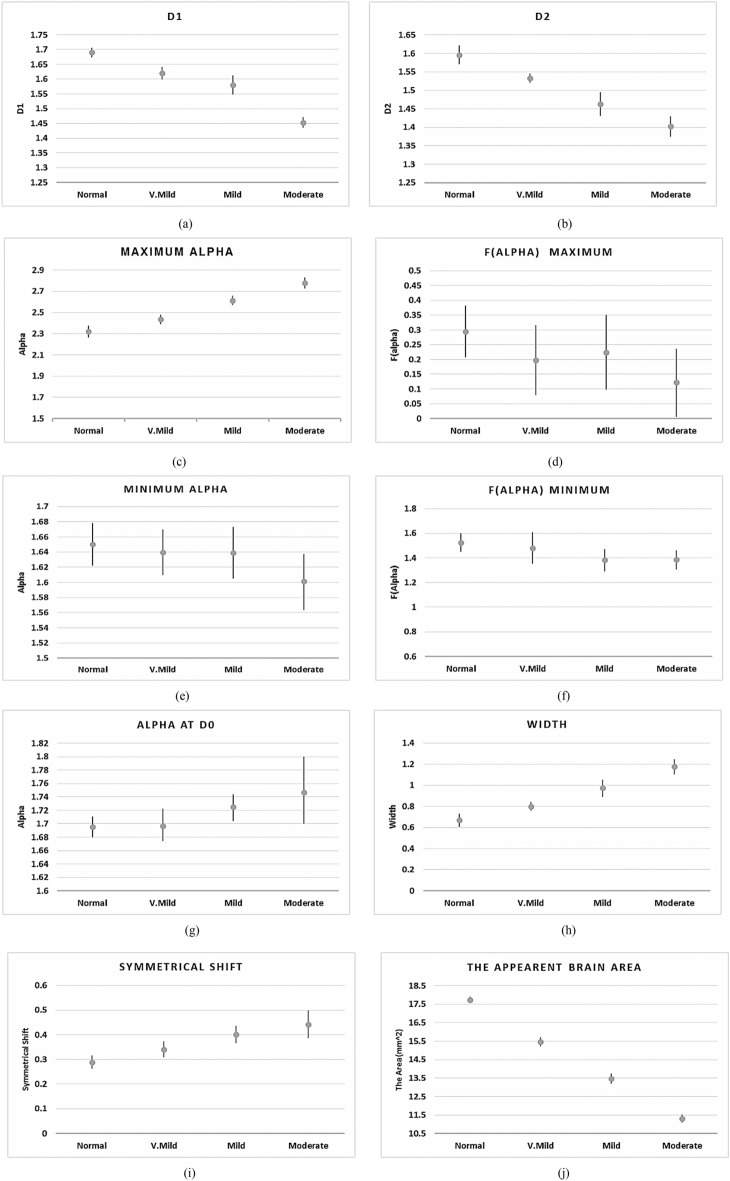
The statistical representation of the extracted features for the AD stages.

The statistical significance of the extracted features using multifractal analysis can be represented in Table 2 using the ANOVA (Analysis of Variances) test. Table 2 shows the average and standard deviation of the extracted features for each AD stage according to the working dataset. According to these results, since the *P* value for all parameters (*P* < 0.05), then all the suggested features have a high significance in the detection of AD. These results in Table 2 can be more clearer as shown in Fig. 13 for the Kaggle dataset as an example. More ANOVA assumptions tests can be listed in Table 3 for the used datasets. Table 3 shows the normality and homogeneity of variance tests for the selected features of the used datasets. The normality test can be achieved by measuring the skewness and kurtosis. The skewness measures the asymmetry of the probability distribution, the distribution could be consistent with a normal distribution if the skewness is between − 2 and + 2. The kurtosis measures whether the samples are around the mean of the distribution or not, the distribution could be consistent with a normal distribution if the kurtosis is between − 2 and + 2. As shown in Table 3, the used features have a normal distribution in both Kaggle and ADNI datasets. The homogeneity of variances can be measured by the *P* value and *F*-test. As shown in Table 3, *P* > 0.05 and *F* value < Fcritical, therefore the null hypothesis can not be rejected, i.e., the features group has homogeneity in variances.

**Table 2:** The statistical significance of the extracted features.

Image	Kaggle dataset				ADNI dataset			*P* value	
	No dementia	Very mild dementia	Mild dementia	Moderate dementia	CN	MCI	AD	Kaggle	ADNI
*D*_1_	1.69 ± 0.016	1.62 ± 0.021	1.58 ± 0.032	1.453 ± 0.018	1.82 ± 0.012	1.76 ± 0.021	1.65 ± 0.03	1e-4	2.3e-4
*D*_2_	1.596 ± 0.025	1.533 ± 0.012	1.463 ± 0.032	1.402 ± 0.028	1.74 ± 0.025	1.65 ± 0.011	1.54 ± 0.024	1e-4	6.2e-4
*α*_max_	2.318 ± 0.055	2.613 ± 0.027	2.635 ± 0.063	2.776 ± 0.052	2.42 ± 0.032	2.62 ± 0.044	2.83 ± 0.036	7.2e-5	3e-4
*f*(*α*_max_)	0.294 ± 0.078	0.19697 ± 0.018	0.224 ± 0.042	0.122 ± 0.014	0.634 ± 0.011	0.435 ± 0.063	0.235 ± 0.034	1e-4	5.2e-4
*α*_min_	1.65 ± 0.028	1.64 ± 0.03	1.634 ± 0.034	1.601 ± 0.037	1.53 ± 0.022	1.568 ± 0.042	1.635 ± 0.027	3.2e-5	1e-4
*f*(*α*_min_)	1.542 ± 0.072	1.481 ± 0.027	1.383 ± 0.097	1.37 ± 0.082	1.21 ± 0.021	1.335 ± 0.066	1.46 ± 0.036	4e-6	1e-4
Spectrum width	0.67 ± 0.062	0.796 ± 0.042	0.974 ± 0.079	1.175 ± 0.069	0.935 ± 0.071	1.124 ± 0.068	1.436 ± 0.056	5.2e-5	1e-4
*α*_0_	1.69 ± 0.016	1.702 ± 0.022	1.724 ± 0.019	1.75 ± 0.049	1.623 ± 0.032	1.733 ± 0.023	1.832 ± 0.019	2.3e-4	1e-4
Symmetrical shift	0.289 ± 0.027	0.341 ± 0.032	0.401 ± 0.035	0.442 ± 0.042	0.211 ± 0.041	0.334 ± 0.025	0.452 ± 0.015	1e-4	3.3e-4
*A*(mm^2^)	17.71 ± 0.158	15.46 ± 0.151	13.47 ± 0.148	11.31 ± 0.207	18.63 ± 0.132	16.32 ± 0.169	14.58 ± 0.201	3.6e-6	6.3e-4

**Table 3:** ANOVA assumptions for the extracted features.

Image	Kaggle dataset				ADNI dataset			
	Normality		Homogeneity of variances		Normality		Homogeneity of variances	
	Skewness	Kurtosis	*P* value	*F*-test (1.394)	Skewness	Kurtosis	*P* value	*F*-test (0.8)
*D*_1_	0.352	− 0.689	0.639	1.35	0.251	− 0.425	0.526	0.623
*D*_2_	0.638	− 0.962	0.82	0.62	− 0.325	0.468	0.63	0.26
*α*_max_	0.147	− 1.19	0.75	1.098	0.542	− 1.02	0.254	0.531
*f*(*α*_max_)	− 0.626	0.44	0.084	1.236	− 0.371	0.36	0.753	0.206
*α*_min_	− 0.26	− 0.42	0.957	0.105	1.32	− 0.655	0.238	0.124
f(α_min_)	0.212	− 0.658	0.324	1.21	0.623	− 0.745	0.759	0.546
Spectrum width	0.243	− 1.11	0.09	1.268	0.124	− 0.368	0.332	0.314
*α*_0_	− 0.618	1.37	0.344	1.234	− 0.854	0.214	0.572	0.726
Symmetrical shift	0.682	1.77	0.379	1.04	− 0.773	0.925	0.222	0.635
*A*(mm^2^)	0.034	− 1.29	0.34	1.0304	0.525	− 0.105	0.348	0.542

where, *F*_critical_ = 1.394 in case of Kaggle dataset and *F*_critical_ = 0.8 in case of ADNI dataset according to *F*_critical_ tables.

Figure 13 shows the statistical representation of the extracted features for the AD stages. As the brain atrophy increased, the generalized dimensions *D*_1_ and *D*_2_ as in Fig. 13a,b have a significant change due to more gaps and a change in the structure of the brain. In Fig. 13b,c, as the deposition of beta-amyloid and total tau (*T*-tau) increased, the AD singularity spectrum curves shift to right with increasing in the maximum local dimension (*α*_max_) value, with decreasing the singularity spectrum end f(α_max_) due to the dementia stage . In Fig. 13e,f, the minimum local dimension (*α*_min_) and *f*(*α*_min_) have reached their maximum values in normal stage with decreasing until reaching the moderate stage. As in Fig. 13g, the local dimension (*α*_0_) has reached its minimum value in normal stage with tendency to increase. So, there are a significant changes in the spectrum width and the symmetrical shape according to the AD stages, this is illustrated in Fig. 13h,i respectively. As the brain regions begin to shrink due to the neurons die and connections breakdown, the brain apparent area will be decreased as shown in Fig. 13j.

In order to automate the classification of the Alzheimer's stages, a classification system based on a simple KNN can be used. The use of the simple classifier model is due to the presence of a noticeable discrepancy in the data extracted from the images by using multifractal geometry, which facilitates the classification process for any classification model. In addition, there is no longer the need to have large data or use many extracted features to express a structural change in the brain.

For measuring the proposed classification system quality, the first classification system uses the Kaggle dataset, 71% of the dataset is used for training, while 29% of the dataset is used for testing. As mentioned before, for 160 MRI images in each stage, 100 images for training and 40 images for testing. While the second classification system uses the ADNI dataset, with 80% of the data for training and 20% for testing. Table 4 to Table 7 summarize the resulting performances, the reported metrics are the average of 7 runs in order to get an accurate result.

12 $$ Sensitivity \left( \% \right) = \frac{TP}{{TP + FN}} \times 100 $$

13 $$ Specificity \left( \% \right) = \frac{TN}{{TN + FP}} \times 100 $$

14 $$ Precision \left( \% \right) = \frac{TP}{{TP + FP}} \times 100 $$

**Table 4:** The classification data for Kaggle dataset.

Item	Normal	Very mild	Mild	Moderate	Total
Tested images	40	40	40	40	160
Correctly classified	40	40	39	40	159
Incorrectly classified	0	0	1	0	1
Classification accuracy	100%	100%	97.5%	100%	99.4%

**Figure 14:**
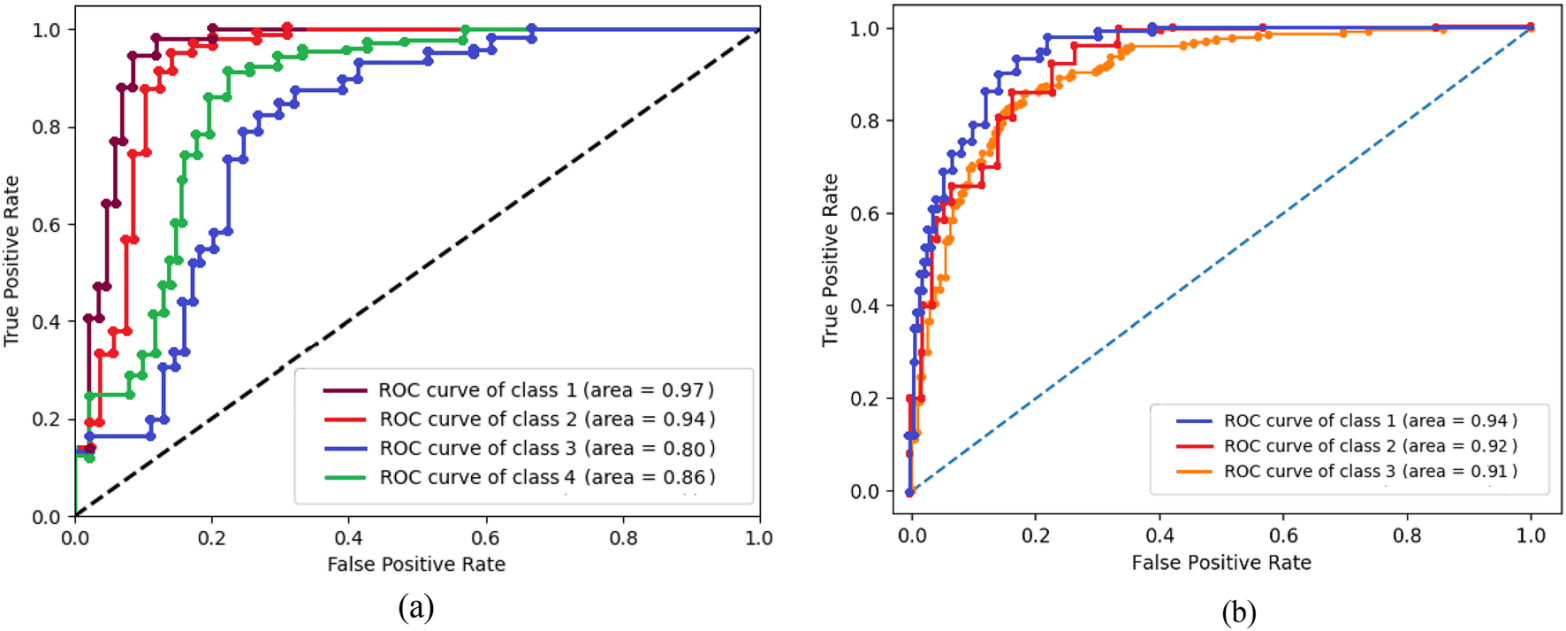
The ROC curves for the four AD classes (**a**) Kaggle dataset (**b**) ADNI dataset.

The Receiver Operator Characteristic (ROC) curve and the Area Under Curve (AUC) can be calculated from Fig. 14, hence the AUC is the ability for distinguishing between the AD stages. In Fig. 14a, in using the Kaggle dataset class 1 (No-dementia) has AUC with 0.97, class 2 (very mild dementia) has 0.94 AUC, class 3 (mild dementia) has 0.80 AUC, and class 4 (moderate dementia) has 0.86 AUC. Figure 14 (b) for the ADNI dataset, class1 (Normal) has AUC with 0.91, class 2 (MCI) has 0. 92 AUC, and class 3 (AD) has 0.91 AUC.

From Tables 4, 5, 6, 7, the proposed classification system has achieved a classification accuracy of 99.4%, sensitivity of 100%, 98.89% as an average specificity, and 97.6% as a minimum precision in the case of the first dataset (Kaggle). While in the case of the ADNI dataset, the proposed system has achieved 99.3% accuracy, 100% sensitivity, and 98.65% average specificity. This technique can detect the early stages of AD, especially very mild and mild stages, it can be extended to classify other medical images.

**Table 5:** The classification data for ADNI dataset.

Item	CN	MCI	AD	Total
Tested Images	50	50	50	150
Correctly classified	50	50	49	149
Incorrectly classified	0	0	1	1
Classification accuracy	**100%**	**100%**	**98%**	**99.3%**

**Table 6:** performance measures.

	Confusion matrix				Performance parameters (%)		
Actual status	TP	FN	FP	TN	Sensitivity	Specificity	Precision
Normal (40) vs. Pathological (120)	40	0	1	119	100	99.17	97.6
V.Mild (40) vs. Mild (40)	40	0	1	39	100	97.5	97.6
V.Mild (40) vs. Moderate (40)	40	0	0	40	100	100	100

where, *TP* true positive, *TN* true negative, *FN* false negative, *FP* false positive.

### Comparative analysis

To ensure the effectiveness of the proposed classification system, a comparative analysis with other classification techniques has been introduced in Table 8. As illustrated in Table 8, several AD stages were classified according to the classification techniques. The research that were compared with our proposed methodology can be divided into three categories:

**Table 8:** Classification techniques evaluation.

Reference	Method	AD stages	Accuracy (%)	Sensitivity (%)	Precision (%)	Specificity (%)	datasets
L. Bloch et. al. ^58^	eXtreme Gradient Boosting (XGBoost) and RF	CN and MCI	71.03	–	–	–	1700 (ADNI) and 612 (AIBL) subjects
F. Al-Khuzaie et. al. ^59^	CNN (Alzheimer network)	CN and AD	99.3	–	98.92	–	15,200 MRI slices
Hamed et. Al ^60^	CNN	CN, EMCI and LMCI	94.54	91.7	–	98.19	The MRIs of 3600 individuals
M. Rohini et. al ^61^	Multivariate linear regression, LR, and SVM	CN, AD, and MCI	89	–	–	–	1000 MRI baseline assessment data from (ADNI)
S. Salunkhe et. al ^62^	Gray level Co-occurrence matrix (GLCM) and 20 features texture classification	CN and AD	Ensemble (90.2%), Decision Trees (88.5%), and (SVM) (87.2%)	–	–	–	MRI ADNI database
Ranjbar, S et. al ^63^	Diagonal quadratic discriminant analysis and NB	CN, AD and MCI	89	82	–	87	173 unique patients in ADNI database
Y. Huang1,11, et al.^64^	EWASplus based RF, LR, SVM and decision tree	CN, and AD	96.2	–	85.8	–	717 samples from ROS/MAP cohort
N. J. Herzog et. al ^65^	KNN, SVM, Linear Discriminant (LD), NB and CNN	CN, EMCI and AD	93	98	–	95	600 MRI of ADNI database
N.M. Khan et. Al. ^66^	VGG architecture	CN, AD and MCI	99.2	99.5 max	–	99.4	2560 MRI ADNI dataset
R. Liu et. al ^67^	local structure preservation sparse representation classifier (LMLS-SRC)	No dementia , V.Mild, Mild, Moderate	85.54	86.19	86.15	84.51	1000 MRI sample of Kaggle datasets
M. Orouskhani et. al ^68^	deep triplet network	No dementia , V.Mild, Mild, Moderate	99.41	85.2	–	–	382 MRI sample of Kaggle datasets
S. Liang et. al ^69^	a WSL-based deep learning	No dementia , V.Mild, Mild, Moderate	98.7	98	99	99.5	6400 MRI sample of Kaggle datasets
H. Ni et. al ^48^	Multifractal applications to resting state functional MRI (rs- MRI) with SVM MKL	CN, and AD	76	90.91	–	79.41	25 AD patients and 38 control from ADNI database
P. Rohinia et. al ^49^	Multifractal and SVM	EMCI, MCI, LMCI, and AD	96.4	96	–	95.7	1055 MRI of ADNI database
**The proposed Algorithm**	**Multifractal and KNN**	**CN, MCI and AD**	**99.3**	**100**	**98**	**98–99.3**	**750 MRI of ADNI database**
	**Multifractal and KNN**	**No dementia, V.Mild, Mild, Moderate**	**99.4**	**100**	**97.6–100**	**97.5–100**	**560 MRI sample of Kaggle datasets**

where, *EMCI* early mild cognitive impairment, *LMCI* late mild cognitive impairment, *NB* naïve bayes, *LR* logistic regression, *RF* random forest, *MKL* multiple kernel learning.

The first category contains the researches that used different traditional or modified classification techniques as in^58–66^. In^59,60^, they used CNN as a classifier with an accuracy of 99.3% and 94.54% respectively, taking into consideration that the methodology in^59^ is used for binary classification not for multi-stage classification. Several classification techniques were embedded in^64^ as EWASplus-based RF, LR, SVM, and decision tree with 96.2% accuracy and 85.8% precision. While in^65^, they used KNN, SVM, Linear Discriminant (LD), NB, and CNN with 93% accuracy, 98% sensitivity, and 95% specificity. The eXtreme Gradient Boosting (XGBoost) and RF techniques were used^58^ with 71.3% accuracy. In^61–63^, the authors used multi-classification techniques with accuracy 89%, 90.2% max accuracy and 89% respectively. Using VGG network architecture in^66^, the accuracy reached 99.2% with 99.5% as maximum sensitivity.

In the second category, the researches used the same working Kaggle dataset as in^67–69^. The authors in^68^ have achieved 99.41% accuracy, an increase of about 0.11% over the accuracy of the proposed system, while the proposed system has achieved increases in sensitivity by about 15%.

The final category contains the researches that used multifractal geometry as an analysis tool in^48,49^. As a result, the proposed classification methodology has achieved higher performance measures in the classification of AD stages.

## Conclusion

As is well-known, the great challenge in biomedical physics and engineering is the non-invasive assessment of the physiological changes to happen inside the human body. Concerning AD, early detection can survive the patients' lives from deterioration of the disease. Therefore, to improve the classification of AD, the two major contributions in current work are focused on the automated multiclass diagnosis of dementia, in accordance with MRI of the human brain. Those contributions are described as follows:

- As the size of the brain gets shrinks with AD, multifractals analysis has been applied to extract the most vital and essential eight features related to the brain changes.
- The KNN algorithm has been implemented to automate the classification process to assign the patient to one of four categories: no cognitive decline, very mild cognitive decline, mild cognitive decline, and moderate cognitive decline.

Remarkably, this new promising approach is very simple, robust and consists of four main steps, namely, image acquisition, preprocessing, feature extraction, and classification. Multifractals enable feature reduction compared with alternate extracting features algorithms. The classification methodology has achieved 99.4% of accuracy for the Kaggle dataset and 99.3% for the ADNI dataset. Moreover, the sensitivity, precision, and specificity have reached up to 100%. The proposed technique has been tested and compared with different approaches concerning the early detection of AD disease. It is easy to note the strength of the proposed model, which produces accurate, fast, and reliable, results as well as the best candidate for applicable. Concluding, it is indeed sensible and of great significance to integrate multifractals analysis and machine learning methods in biomedical physics and engineering research.

## Data Availability

The datasets were collected from Kaggle international data science community, and ADNI datasets. The datasets are available in: https://www.kaggle.com/datasets/tourist55/alzheimers-dataset-4-class-of-images, https://adni.loni.usc.edu/data-samples/access-data/.
